# Unsupervised learning of interacting topological phases from experimental observables

**DOI:** 10.1016/j.fmre.2022.12.016

**Published:** 2023-01-20

**Authors:** Li-Wei Yu, Shun-Yao Zhang, Pei-Xin Shen, Dong-Ling Deng

**Affiliations:** a Theoretical Physics Division, Chern Institute of Mathematics and LPMC, Nankai University, Tianjin 300071, China; b Center for Quantum Information, IIIS, Tsinghua University, Beijing 100084, China; c Shanghai Qi Zhi Institute, 41th Floor, AI Tower, No. 701 Yunjin Road, Xuhui District, Shanghai 200232, China

**Keywords:** Unsupervised learning, Topological phases, Diffusion map, Spectral function, Ultracold atom

## Abstract

Classifying topological phases of matter with strong interactions is a notoriously challenging task and has attracted considerable attention in recent years. In this paper, we propose an unsupervised machine learning approach that can classify a wide range of symmetry-protected interacting topological phases directly from the experimental observables and without a priori knowledge. We analytically show that Green’s functions, which can be derived from spectral functions that can be measured directly in an experiment, are suitable for serving as the input data for our learning proposal based on the diffusion map. As a concrete example, we consider a one-dimensional interacting topological insulators model and show that, through extensive numerical simulations, our diffusion map approach works as desired. In addition, we put forward a generic scheme to measure the spectral functions in ultracold atomic systems through momentum-resolved Raman spectroscopy. Our work circumvents the costly diagonalization of the system Hamiltonian, and provides a versatile protocol for the straightforward and autonomous identification of interacting topological phases from experimental observables in an unsupervised manner.

## Graphical abstract

**Figure:**
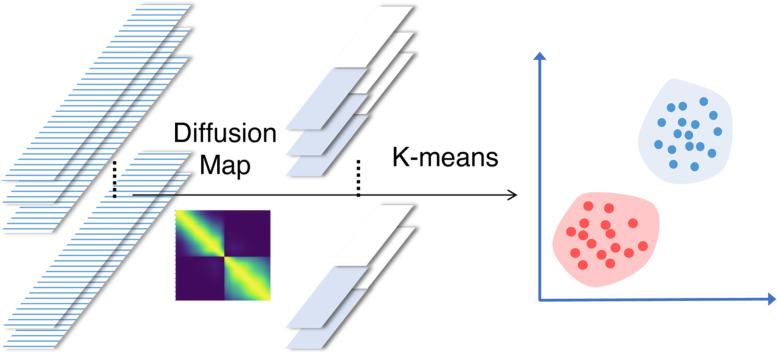

## 1 Introduction

**Fig. 1:**
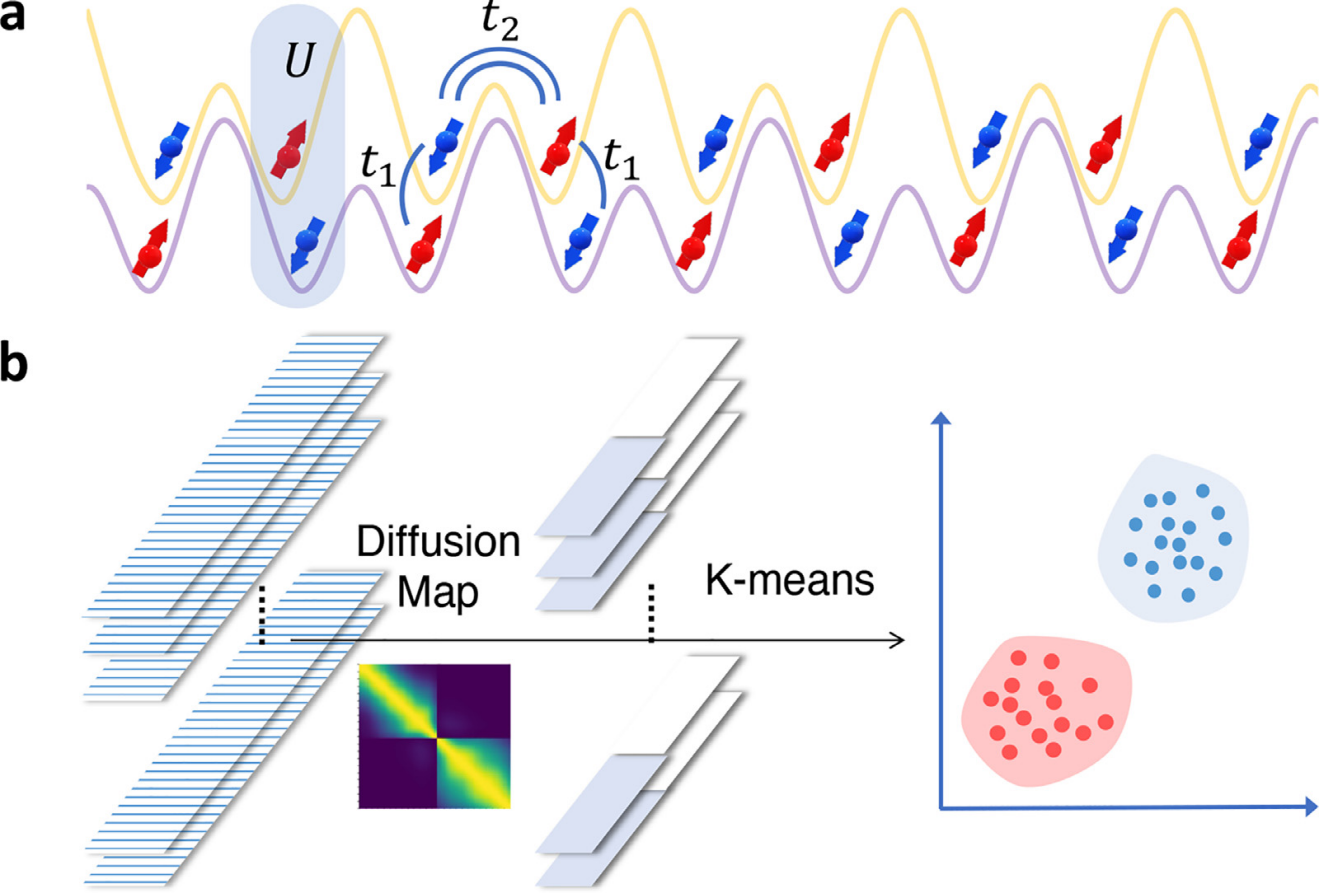
(a) A schematic implementation of the 1D topological insulator with strong interactions in the ultracold atomic gases in optical lattices [27]. (b) Higher dimensional input data samples are non-linearly reduced into the lower dimensional space through the diffusion map method, and then lower dimensional samples are clustered together based on the unsupervised *K*-means method.

In many-body systems, strong interactions between electrons give rise to a variety of striking physical phenomena, including the high-temperature superconductivity [1], [2], Mott transition [3], and fractional quantum Hall effect [4]. On the other hand, the study of topological insulators (TIs) and superconductors [5], [6] is lying at the forefront of modern condensed matter physics. For topological insulators (TIs) with strong interactions [7], considerable efforts have been devoted to unveiling their underlying physics, both in theoretical [8], [9], [10], [11], [12], [13], [14], [15], [16], [17], [18], [19], [20], [21], [22], [23], [24], [25], [26], [27] and experimental aspects [27], [28], [29], [30], [31]. In classifying such interacting topological phases, one significant approach is to extend the topological invariants from non-interacting systems into their interacting counterparts based on the Green’s function method [32]. Along this line, exciting progress has been made, including the construction of topological invariants via Green’s functions [10], [11], [12], [13], simplified zero-frequency Green’s functions for interacting topological systems [14], proposition of topological Hamiltonian [15], and so on. In terms of experimental implementations, quantum simulations of topological insulators with strong on-site interactions have been proposed in ultracold atomic gases trapped in optical lattices [27], [28], [29], [30]. Yet, in sharp contrast to the non-interacting TIs with well-established classification theories [33], a more comprehensive understanding and classification of interacting topological phases remain an ongoing task [7]. Here, we introduce an unsupervised machine learning approach that can identify interacting topological phases directly from experimental observables (see Fig. 1 for a schematic illustration).

Machine learning techniques are powerful in identifying hidden patterns of complex data, and have recently been applied in various contexts of quantum physics [34], [35], ranging from high energy physics [36], quantum dynamics [37], quantum technologies [38], [39], [40], [41], to condensed matter physics [42], [43], [44]. In machine learning topological phases of matter, a variety of approaches have been carried out, including both the supervised [45], [46], [47], [48], [49], [50], [51], [52], [53], [54], [55], [56] and unsupervised ones [56], [57], [58], [59], [60], [61], [62], [63], [64], [65], [66], [67], [68], [69], [70], [71], [72], [73], [74]. In comparison with the supervised learning, one advantage of the unsupervised learning is that it requires no a priori knowledge about the system, and hence is more powerful in detecting those unknown phases. One appealing unsupervised approach is based on the diffusion map [75], [76], [77], which captures the topological and geometrical structures of the data samples through the non-linear dimensionality reduction, and thus is in particular suitable for identifying topological phases [57], [58], [59], [60], [61], [62], [63]. However, most of the above-mentioned works target the non-interacting systems, while rarely focusing on the interacting ones [55], [56], [63]. Besides, choosing experimentally accessible data as the input would greatly improve the applicability of machine learning methods in tackling actual physical systems.

In this paper, we propose to utilize the diffusion map method to classify the interacting topological phases straightforwardly from the experimental observables in an unsupervised manner. Instead of choosing the unit Hamiltonian or Bloch wavefunctions as the input merely for non-interacting systems in the previous works [57], [58], [59], [60], here we choose Green’s function, whose corresponding spectral functions can be directly obtained from experimental observables, as the input data. As a well-established theoretical technique, the Green’s function theory has been shown suitable for constructing topological invariants for a wide range of symmetry-protected topological phases, both with and without interactions [12], [13], [14], [15]. We find that Green’s function can be employed as the input raw data for the phase classification of interacting topological insulators. Then through the theoretical analysis and numerical simulation of the 1D interacting topological insulator, we rigorously show that the dataset of single-particle Green’s functions (spectral functions) is sufficient for the unsupervised learning of interacting topological phases. In what follows, we propose an experimental protocol to measure the spectral function of the 1D interacting topological insulator in the ultracold atomic system using the momentum-resolved Raman spectroscopy [78], [79], [80].

## 2 Theoretical analysis

The diffusion map method provides a non-linear kernel approach to cluster samples based on local similarity. Given a collection of input samples $D=\{x^{\left(1\right)},x^{\left(2\right)},\ldots ,x^{\left(L\right)}\}$, one can describe the local similarity between two samples $x^{\left(l\right)}$ and $x^{\left(l^{\prime }\right)}$ by the Gaussian kernel function $K_{l,l^{\prime }}$ with the variance controlled by the hyper-parameter ϵ (0<ϵ≪1) $K_{l,l^{\prime }}=\exp\left(-∥x^{\left(l\right)}-x^{\left(l^{\prime }\right)}{∥}_{L_{2}}^{2}/\left(2\epsilon \right)\right)$, where $∥x^{\left(l\right)}-x^{\left(l^{\prime }\right)}{∥}_{L_{2}}$ denotes the Euclidean distance between the samples. Define $P_{l,l^{\prime }}=K_{l,l^{\prime }}/\left(\sum_{l^{\prime }}K_{l,l^{\prime }}\right)$ as the one-step diffusion probability from samples l to $l^{\prime }$. Then after 2t steps of diffusion, the diffusion probability from j to $j^{\prime }$ is $D_{t}^{2}\left(j,j^{\prime }\right)=\sum_{k}\lambda_{k}^{2t}{\left[{\left(\psi_{k}\right)}_{j}-{\left(\psi_{k}\right)}_{j^{\prime }}\right]}^{2}$, where $\lambda_{k}$ denotes the kth largest eigenvalue of the diffusion matrix P with the corresponding right eigenvector $\psi_{k}$. Owing to the ergodicity of such diffusion process in the long-time limit [64], [77], the largest eigenvalue of P exactly equals 1. As a consequence, when t→∞, only the largest few components with $\lambda_{k}\approx 1$ dominate in $D_{t}^{2}$. Then the samples are dimensionally reduced and clustered into n categories, with n equal to the number of eigenvalues $\lambda_{k}\approx 1$.

The success of the diffusion map method relies crucially on the input data samples. Within the vein of unsupervised learning non-interacting topological quantum phases, previous works considered a collection of Bloch wave-functions (or equivalently, bulk Hamiltonian vectors) as the input data [57], [58], [59], [66], since these single-particle wave functions encode the full topological information of the models. Yet, the above approach cannot be directly generalized to the interacting systems, especially for systems where a band structure description fails. One alternative approach is to utilize the single-particle Green’s function as the input for learning those interacting topological phases.

As a well-established theoretical technique, the Green’s function method provides a comprehensive approach to defining topological invariants for a wide range of topological insulators, including both the non-interacting and interacting ones. Starting from the momentum space of Green’s function G(Ω,k), one can construct the integral formulas of the topological invariants for topological models in arbitrary spatial dimensions for all ten symmetry classes, in which case the bulk-boundary condition still persists. As an example, let us consider the generalized Thouless–Kohmoto–Nightingale–den Nijs (TKNN) invariant for interacting 2D insulators, where the topological invariant can be expressed as [14]

(1) $$N_{2}=\frac{1}{24\pi^{2}}\int d\Omega d^{2}k\text{Tr}\left[\epsilon^{\mathrm{abc}}G\partial_{a}G^{-1}G\partial_{b}G^{-1}G\partial_{c}G^{-1}\right]$$

where G≡G(iω,k) denotes the time-ordered Green’s function in frequency-momentum space and a, b, c ∈{Ω, $k_{x}$, $k_{y}\}$, with Ω=iω denoting the imaginary Matsubara frequency.

We see from Eq. 1 that the frequency-momentum Green’s function G(iω,k) encodes the full topological information of the phases. However, one major limitation is that such integral formula requires information about Green’s function at all frequencies, which is usually challenging to obtain. It was later shown that G(iω≠0,k) is non-singular for a variety of interacting topological insulators, and the topological invariant can be obtained solely from zero-frequency Green’s functions G(0,k) [14], [15]. This motivates us to choose the zero-frequency Green’s function as the input data for the machine learning of those topological phases.

Without loss of generality, we consider the spinful topological lattice models with the periodic boundary condition, where the momentum space of zero-frequency Green’s function G(0,k) becomes a 2×2 matrix. For convenience, we express its inverse formula (up to an identity term) as $G^{-1}\left(0,k\right)=\vec{g}\left(0,k\right)\cdot \vec{\sigma }$, where the Pauli matrices $\vec{\sigma }=\left(\sigma_{x},\sigma_{y},\sigma_{z}\right)$ span the spin space, and $\vec{g}\left(0,k\right)=\left(g_{x},g_{y},g_{z}\right)$ represents the vector formula of the inverse Green’s function in the spin space.

Now we analytically show that the zero-frequency Green’s functions can be utilized as the input data for learning phases based on the diffusion map method. We choose the input data sample to be $x^{\left(l\right)}=\left\{\hat{g}\left(0,k\right)|k\in \text{BZ}\right\}$, where the unit vector $\hat{g}=\vec{g}/{∥\vec{g}∥}_{L_{2}}$ and BZ denotes the first Brillouin zone. Then one can generate a set of samples $\left\{x^{\left(l\right)}\right\}$ by varying the model parameters $\vec{t}=\left(t_{1},t_{2},\ldots \right)$ contained implicitly in $\hat{g}$ with a constant step $\delta \vec{t}={\vec{t}}^{\left(l+1\right)}-{\vec{t}}^{\left(l\right)}$. By adjusting the hyperparameter ϵ to be $\epsilon ∼∥\delta \vec{t}{∥}_{L_{2}}^{2}$, one obtains that the prominent contributions of the one-step diffusion processing lie between the nearest samples $x^{\left(l\right)}$ and $x^{\left(l+1\right)}$. In the case of small $\delta \vec{t}$, the Gaussian kernel term between the nearest samples can be approximately expressed as $K_{l,l+1}=\exp\left(-∥\nabla_{\vec{t}}x^{\left(l\right)}\cdot \delta \vec{t}{∥}_{L_{2}}^{2}/\left(2\epsilon \right)\right)$, therefore the connectivity between the nearest samples labeled by (l,l+1) depends on the gradient term $\nabla_{\vec{t}}\hat{g}\left(0,k\right)=\nabla_{\vec{t}}(\vec{g}/∥\vec{g}{∥}_{L_{2}})$ for all k∈BZ. It is no doubt that when the gap of $G^{-1}\left(0,k\right)$ closes, i.e., $∥\vec{g}{∥}_{L_{2}}=0$, the gradient term would typically diverse, and the diffusion probability between the two nearest samples divided by the gap closure points of $G^{-1}$ should approximate to zero. Together with the fact that only the diffusion probability of the nearest samples dominates, hence the gap closure points divide the diffusion matrix into blocks. In combination with the fact that when the topological phase transition occurs, eigenvalues of the inverse Green’s function $G^{-1}\left(0,k\right)$ usually equal zero. Thus the data samples belonging to the same topological phase should be connected in the same block of the kernel matrix, and hence be clustered together through the diffusion map method.

The time-ordered Green’s function is not an experimental observable and thus cannot be directly measured. To obtain the aforementioned input data straightforwardly from the experiments, here we use the spectral functions that contain all the information of the time-ordered Green’s function [81]. In the linear response regime, the spectral functions can be directly revealed from the experimental measurement. Once obtaining the spectral functions from the experiment, we can easily obtain the time-ordered Green’s function through $G\left(i\omega ,k\right)=\int d\omega^{\prime }\frac{f(\omega^{\prime })}{i\omega -\omega^{\prime }}$, where $f(\omega^{\prime },k)$ means the spectral function. In the last part of this work, we propose an experimental scheme to implement the interacting topological models in the ultracold atomic system and measure the corresponding spectral functions using the momentum-resolved Raman spectroscopy [78], [79], [80].

## 3 The model

To illustrate how our approach works, we consider the following 1D interacting topological insulator model with the Hamiltonian [27]

(2) $$\begin{array}{ccc} \hat{H} & = & -\sum_{i=1}^{N}\left(t_{1}c_{i,↑}^{†}c_{i,↓}+t_{2}c_{i,↑}^{†}c_{i+1,↓}+h.c.\right)\\ & & +U\sum_{i=1}^{N}\left(n_{i,↑}-\frac{1}{2}\right)\left(n_{i,↓}-\frac{1}{2}\right) \end{array}$$

where $t_{1}$ is the spin-flip amplitude, $t_{2}$ is the hopping amplitude, U denotes the strength of the on-site interaction, N denotes the size of the model, i represents the index of the site, $c_{i,\sigma }^{†}\left(c_{i,\sigma }\right)$ is the creation (annihilation) fermionic operator with spin σ={↑,↓}, and $n_{i,\sigma }=c_{i,\sigma }^{†}c_{i,\sigma }$ represents the fermionic particle number operator. The above interacting model may be realized in an ultracold atomic system [27]. Such model hosts the chiral symmetry $\hat{\Sigma }$, i.e., $\hat{\Sigma }\hat{H}{\hat{\Sigma }}^{†}=\hat{H}$, with the symmetry operator $\hat{\Sigma }=\prod_{j}\left[c_{j,↑}^{†}+{\left(-1\right)}^{j}c_{j,↑}\right]\left[c_{j,↓}^{†}+{\left(-1\right)}^{j}c_{j,↓}\right]$. Accordingly, for the Hamiltonian with chiral symmetry, the corresponding Green’s function G(iω,k) satisfies the condition CG(iω,k)C=−G(−iω,k) [17], where the chiral operator C squares to 1. In our bases with G(iω,k) having the off-diagonal structure, the chiral operator takes the standard formula $C=\left(\begin{array}{cc} 1 & 0\\ 0 & -1 \end{array}\right)$. In the non-interacting limit U=0, the Hamiltonian turns equivalently into the 1D Su–Schrieffer–Heeger (SSH) model, which belongs to the BDI symmetry class with a Z topological classification [33], [82]. Here, to validate the capability of our unsupervised learning approach with Green’s functions as input, we apply our learning method to the phase classification of the 1D SSH model, and successfully locate the corresponding topological phase boundary with a high precision, see ref. [83] for details.

## 4 Learning phases with strong interactions

**Fig. 2:**
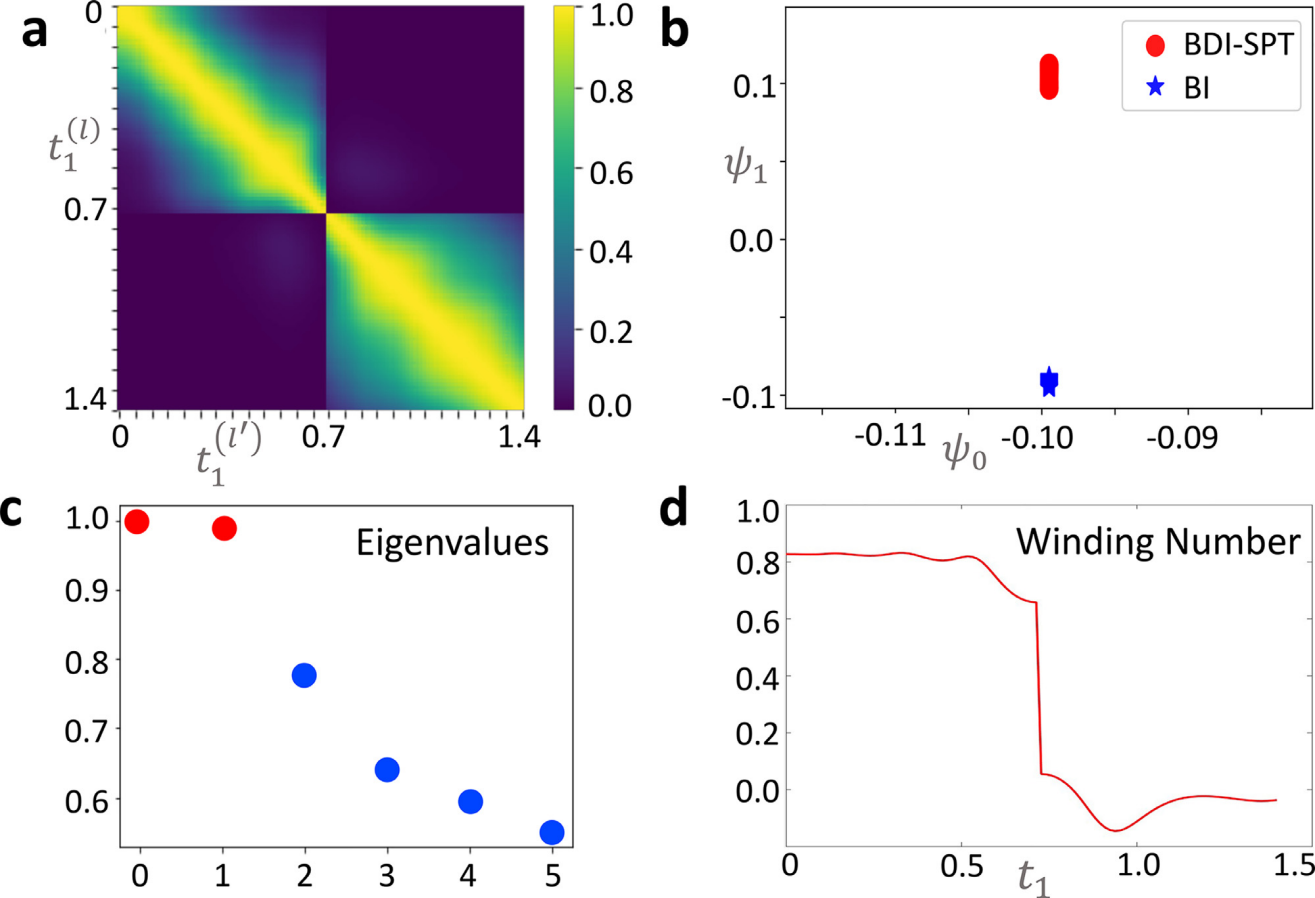
**Machine learning phases of the interacting topological insulator with the data set of Green’s function.** (a) Heatmap of the Gaussian kernel value between the data samples with varying $t_{1}$. (b) Scatter diagram of the two eigenvectors $\left\{\psi_{0},\psi_{1}\right\}$ of the diffusion matrix with the largest two eigenvalues λ≈1. The samples are clustered into two phases, where the red circle denotes the BDI-SPT phase, and the blue star denotes the band insulator (BI) phase. (c) The leading eigenvalues of the diffusion matrix. (d) Winding number obtained from the numerically simulated spectral function with varying $t_{1}$. Parameters: number of sites N=6, the variance parameter ϵ=0.003, $t_{2}=1$, U=1, the varying parameter $t_{1}^{\left(l\right)}=0.014*\left(l-1\right)$ for each sample $x^{\left(l\right)}$, with l∈[1,101].

Let us now consider the case of U>0 in Eq. 2, where the presence of repulsive on-site interacting terms modifies the original non-interacting phase boundary $t_{1}=t_{2}$ [27]. To learn the phase boundaries of the interacting SSH model in an unsupervised manner, we choose Green’s functions as the input data set, i.e., $\left\{x^{\left(l\right)}|x^{\left(l\right)}=\left[{\hat{g}}^{\left(l\right)}\left(0,k\right),k\in \left[-\pi ,\pi \right)\right]\right\}$ by varying the spin flipping strength $t_{1}$ from 0 to 1.4, while fixing the hopping strength $t_{2}=1$, and the on-site interaction strength U=1. ${\hat{g}}^{\left(l\right)}\left(0,k\right)$ can be obtained from the spectral functions A(ω,k). Through the diffusion map method, we show our numerical results in Fig. 2a–c. We see from Fig. 2a that the Gaussian kernel matrix K is separated into two blocks, indicating that the corresponding diffusion matrix P has two largest eigenvalues $\lambda_{0,1}\approx 1$, as shown in Fig. 2c. Consequently, the set of samples is classified into two topological phases. The clustering result can be clearly observed from the scatter diagram of the two eigenvectors $\left\{\psi_{0},\psi_{1}\right\}$ in Fig. 2b. Hence we see that the input samples are automatically clustered into two categories and the learned phase boundary locates at $t_{1}\approx 0.721$. In Fig. 2d, we show that a 40×6 pixelization of (ω,k) yields the winding number χ≈0.8 (χ≈0.1) for the topological non-trivial (trivial) case, which deviates from the theoretical integer winding number χ=1 (χ=0). Nevertheless, the predicted phase boundary based on both the diffusion map and the topological invariant coincides exactly with the phase boundaries predicted in Ref. [27]. This indicates the robustness of our learning method against the discretization of the frequency and the momentum. As a result, one labels the red circle samples in Fig. 2b by the topological non-trivial phase, and the blue star samples in Fig. 2b by the topological trivial phase, respectively.

## 5 Measuring the spectral functions

The interacting SSH model considered in Eq. 2 may be realized with ultracold fermionic atoms in the spin-dependent 1D optical superlattices [27], where the spin-flip hopping $t_{1}$ can be implemented by the Rabi coupling through an external laser light, the parameter $t_{2}$ can be implemented by the hopping in a double-well potential, and the interacting strength U is realized by the on-site s-wave scattering interaction of two fermionic atoms with different internal (spin) components, see Fig. 1a. Here, we mainly focus on the detection of the spectral functions of such an interacting system, which is assumed to be prepared in its ground state. In ultracold atomic gases, one can accomplish such detection through the momentum-resolved Raman [78], [79], [80] or radio-frequency spectroscopy [84]. We denote the fermionic atoms with two internal (spin) states by $\{|\alpha \rangle ,|\alpha^{\prime }\rangle \}$, and consider another initially unoccupied internal state |β〉. For simplicity, we suppose that the couplings between atoms with internal state |β〉 and the left atoms (in state |α〉, $|\alpha^{\prime }\rangle$ or |β〉) are negligible, i.e., particles with internal states |β〉 have the single-particle dispersion relation for atoms with energy $\varepsilon_{\beta k}$. By adding a pair of laser beams with the wavevector difference $q=k_{1}-k_{2}$ and frequency difference $\omega =\omega_{1}-\omega_{2}$, one transfers the atoms from the $\{|\alpha \rangle ,|\alpha^{\prime }\rangle \}$ states to another internal state |β〉 via the intermediate excited state |γ〉. The beams are far from the resonance with the excited state |γ〉 so that spontaneous emission can be neglected. Then the Raman transfer rate to the state |β〉 with momentum k can be obtained utilizing Fermi’s golden rule and is shown proportional to the spectral function of the atoms in state |α〉 [78], [79], i.e., $\Gamma_{k}\left(\Omega ,q\right)\propto n_{F}\left(\varepsilon_{\beta k}-\hbar \Omega -\mu \right)A_{\alpha }\left(\varepsilon_{\beta k}-\hbar \Omega -\mu ,k-q\right)$, where μ denotes the chemical potential of the system, $n_{F}$ denotes the fermionic distribution function, and $A_{\alpha }=f_{\alpha \alpha }$ denotes the diagonal part of the spectral function matrix $f(\omega^{\prime },k)$ in internal space. At zero temperature, $n_{F}\left(\hbar \omega \right)$ equals zero (unity) for ω>0 (ω<0). One can then reveal the transfer rate $\Gamma_{k}$ from the time-of-flight absorption image of |β〉-state atoms, and thus obtains the k-dependent spectral function. The frequency dependence of the spectral function can be obtained by repeating the different frequencies ω. As a result, one measures the spectral function A(ω,k) with ω<0. Similarly, to measure the spectral function A(ω,k) with ω>0, one utilizes the reversed Raman process, which requires that a large incoherent population of atoms are initially in the state |β〉. Measuring the decrease of the |β〉-state population produces A(ω,k) for ω>0.

With the spectral functions $A_{\alpha }\left(\omega^{\prime },k\right)$ produced from the measurements, we then calculate the diagonal component of Green’s function $G_{\alpha \alpha }\left(i\omega ,k\right)$. A similar procedure produces $A_{\alpha^{\prime }}\left(\omega^{\prime },k\right)$ and $G_{\alpha^{\prime }\alpha^{\prime }}\left(i\omega ,k\right)$. To obtain the off-diagonal component $G_{\alpha \alpha^{\prime }}$, we apply an impulsive pulse right before the photoemission/photoabsorption process to induce a basis rotation between different spin components [85], [86], while preserving the atomic momentum, see ref. [83] for details.

It is worthwhile to mention that our learning method is robust to the experimental imperfections in actual measurements. For instance, it is challenging to obtain the spectral function A(ω,k) for all real values of ω and k owing to the limited experimental resolutions, hence we may only reveal A(ω,k) at discrete momentum and frequency points from the experimental measurements. Thus Green’s function, as the integral of spectral functions, can only be computed approximately after discretization. Besides, an additional harmonic confining potential in trapping the atomic gas should be involved in a typical experimental setup. We consider all of the above cases in our numerical calculations [83], and find that the learned phase boundaries are extremely stable and robust to those imperfections. Another advantage of our learning approach is that it only requires the experimental measurements as the input data without any prior knowledge about the system, hence circumventing the costly full-diagonalization of the system Hamiltonian in real space, which is, in some sense, usually indispensable in obtaining the projective matrix as the input data for machine learning topological phases [59], [60].

## 6 Discussion and conclusion

The physical consequences of strong interactions in topological phases of matter are by no means limited to the interacting topological insulators. Actually, strong interactions would induce more intricate systems, e.g., models with intrinsic topological order, which usually host the long-range entangled groundstates, and anyonic excitations [87]. Classification of the intrinsic topological phases is highly non-trivial. In the future, it would be interesting and desirable to extend our unsupervised method to the identification of intrinsic topological phases.

In summary, we have introduced an unsupervised learning approach that can identify a wide range of interacting topological phases from experimental observables. We analytically show that Green’s function can be utilized as the input data for learning both the interacting and non-interacting phases. Through the numerical simulations, we demonstrate that our approach is robust to those prevalent experimental imperfections, and thus is experimentally feasible. We then propose a generic scheme to measure the spectral functions in ultracold atomic systems through momentum-resolved Raman spectroscopy. Our findings shed new light on further studies of machine learning interacting topological phases of matter, both in theory and experiment.

## Declaration of competing interest

The authors declare that they have no conflicts of interest in this work.

## Appendix A Supplementary materials

Supplementary Data S1 Supplementary Raw Research Data. This is open data under the CC BY license http://creativecommons.org/licenses/by/4.0/ Supplementary Data S1
