# Supplementary material for: Unsupervised learning of interacting topological phases from experimental observables
Source: Fundam Res. 2023 Jan 20;4(5):1086–91. doi: 10.1016/j.fmre.2022.12.016 (PMC11630675; doi:10.1016/j.fmre.2022.12.016)
Supplement: Supplementary Data S1 — Supplementary Raw Research Data. This is open data under the CC BY license http://creativecommons.org/licenses/by/4.0/ [file mmc1.pdf]

Supplementary Materials for: Unsupervised Learning of Interacting Topological Phases from Experimental Observables

Li-Wei Yu,[1,2,*] Shun-Yao Zhang,[2,*] Pei-Xin Shen,[2] and Dong-Ling Deng[2,3,†]

[1] *Theoretical Physics Division, Chern Institute of Mathematics and LPMC, Nankai University, Tianjin 300071, P. R. China*
[2] *Center for Quantum Information, IIIS, Tsinghua University, Beijing 100084, P. R. China*
[3] *Shanghai Qi Zhi Institute, 41th Floor, AI Tower, No. 701 Yunjin Road, Xuhui District, Shanghai 200232, China*

CONTENTS

SI. Green's function method for interacting topological insulators 2
A. Green's function method and spectral functions 2
B. Definition of topological invariants via Green's functions 3

SII. Numerical simulations of the spectral functions 4

SIII. More details about the unsupervised learning of interacting topological phases 5
A. Learning non-interacting topological insulators based on spectral function: The 1D SSH model 5
B. More about the learning results of the 1D interacting topological insulator 6

SIV. Topological invariants from momentum-space Green's function 6
A. The non-interacting case: $U = 0$ 7
B. The interacting case: $U = 1$ 8
C. The interacting case: $U = 4$ 9

SV. Introduction to the diffusion map method 10

References 10

* These authors contributed equally to this work.
† dldeng@tsinghua.edu.cn

SI. GREEN'S FUNCTION METHOD FOR INTERACTING TOPOLOGICAL INSULATORS

A. Green's function method and spectral functions

Green's function method plays important roles in dealing with interacting many-body systems. In this paper, we mainly focus on the single-particle Green's function, with a retarded or advanced formula, as

$$G^r_{i,j}(t) = \theta(t)(i\langle 0|\tilde{T}c_i(t)c_j^\dagger(0)|0\rangle - i\langle 0|Tc_i(t)c_j^\dagger(0)|0\rangle), \text{ (Retarded)} \tag{S1}$$

$$G^a_{i,j}(t) = \theta(-t)(i\langle 0|\tilde{T}c_i(t)c_j^\dagger(0)|0\rangle - i\langle 0|Tc_i(t)c_j^\dagger(0)|0\rangle), \text{ (Advanced)} \tag{S2}$$

where $\theta(t)$ represents the step function of time t, $\theta(t>0)=1$ and $\theta(t<0)=0$, T (or $\tilde{T}$) denotes the time ordered (or anti-time ordered) operator, and $c_i^\dagger$ (or c_i) denotes the fermionic generation (or annihilation) operator on the i-th site. Under the time-frequency Fourier transformation, the retarded (advanced) Green's function can be transformed into the frequency space $G(i\omega)$ with the frequency $\omega>0$ ($\omega<0$).

Supposing the system is in the periodic boundary condition, then we can write the frequency-momentum space Matsubara Green's function with the Lehmann representation (in the zero-temperature limit) as follows,

$$G_{\alpha\alpha'}(i\omega,\mathbf{k}) = \sum_n \left[\frac{\langle\psi_n|c_{\mathbf{k},\alpha}|\psi_0\rangle\langle\psi_0|c^\dagger_{\mathbf{k},\alpha'}|\psi_n\rangle}{i\omega+(E_n-E_0)} + \frac{\langle\psi_0|c_{\mathbf{k},\alpha}|\psi_n\rangle\langle\psi_n|c^\dagger_{\mathbf{k},\alpha'}|\psi_0\rangle}{i\omega-(E_n-E_0)}\right], \tag{S3}$$

where $\{\alpha,\alpha'\}$ represents the internal (spin) label of the system, $|\psi_n\rangle$ represents the eigenstate of the Hamiltonian $\hat{H}-\mu\hat{N}$ with the corresponding eigenvalue E_n, $|\psi_0\rangle$ denotes the ground state with energy E_0; $\hat{H}$ is the many-body Hamiltonian, μ is the chemical potential, and $\hat{N}$ means the total fermionic number of the system.

By defining the corresponding spectral function

$$f_{\alpha\alpha'}(\omega,\mathbf{k}) = \begin{cases} \sum_n \langle\psi_n|c_{\mathbf{k},\alpha}|\psi_0\rangle\langle\psi_0|c^\dagger_{\mathbf{k},\alpha'}|\psi_n\rangle\delta(\omega+E_n-E_0) & \text{for } \omega<0, \\ \sum_n \langle\psi_0|c_{\mathbf{k},\alpha}|\psi_n\rangle\langle\psi_n|c^\dagger_{\mathbf{k},\alpha'}|\psi_0\rangle\delta(\omega+E_0-E_n) & \text{for } \omega>0, \end{cases} \tag{S4}$$

then one can express the Matsubara Green's function in Eq. (S3) into the following spectral function formula

$$G_{\alpha\alpha'}(i\omega,\mathbf{k}) = \int d\omega' \frac{f_{\alpha\alpha'}(\omega',\mathbf{k})}{i\omega-\omega'}. \tag{S5}$$

In this work, we put forward an experimental proposal that can reveal the values of the spectral functions $f_{\alpha\alpha'}(\omega,\mathbf{k})$ from observables based on the momentum-resolved Raman spectroscopy. In our proposal, it is more convenient to detect the diagonal part of the spectral functions $f_{\tau\tau}(\omega,\mathbf{k}) = A_\tau(\omega,\mathbf{k})$ in internal (spin) space, as

$$A_\tau(\omega,\mathbf{k}) = \begin{cases} \sum_n |\langle\psi_n|c_{\mathbf{k},\tau}|\psi_0\rangle|^2\,\delta(\omega+E_n-E_0) & \text{for}\,\omega<0, \\ \sum_n \left|\langle\psi_n|c^\dagger_{\mathbf{k},\tau}|\psi_0\rangle\right|^2\delta(\omega+E_0-E_n) & \text{for}\,\omega>0, \end{cases} \tag{S6}$$

To reveal the non-diagonal part of the spectral function $f_{\alpha\alpha'}$ ($\alpha\neq\alpha'$) in internal (spin) space, we need to measure the spectral function $A_\tau(\omega,\mathbf{k}) =$ along different spin directions. For example, one can measure $A_\tau(\omega,\mathbf{k})$ for $\tau=\{\alpha,\alpha',+,I\}$ assisted by the momentum-preserved spin rotations. To measure A_+ with $|+\rangle = (|\alpha\rangle+|\alpha'\rangle)/\sqrt{2}$, one can apply a $\frac{\pi}{2}$-pulse to couple the internal (spin) states $|\alpha\rangle$ and $|\alpha\rangle$. To measure A_I with $|I\rangle = (|\alpha\rangle - i|\alpha'\rangle)/\sqrt{2}$, one can apply a phase shift $\frac{\pi}{2}$-pulse to couple the internal (spin) states $|\alpha\rangle$ and $|\alpha\rangle$. One can expand the spectral functions A_+ and A_I in the bases $\{\alpha,\alpha'\}$. We first consider the case of $\omega>0$, where

$$\begin{aligned} A_+(\omega,\mathbf{k}) &= \sum_n |\langle\psi_n|c_{\mathbf{k},+}|\psi_0\rangle|^2\,\delta(\omega+E_n-E_0), \\ &= \sum_n \left|\langle\psi_n|\frac{c_{\mathbf{k},\alpha}+c_{\mathbf{k},\alpha'}}{\sqrt{2}}|\psi_0\rangle\right|^2\delta(\omega+E_n-E_0), \\ &= \frac{1}{2}\left[A_\alpha(\omega,\mathbf{k})+A_{\alpha'}(\omega,\mathbf{k})+f_{\alpha\alpha'}(\omega,\mathbf{k})+f_{\alpha'\alpha}(\omega,\mathbf{k})\right], \end{aligned} \tag{S7}$$

and

$$\begin{aligned} A_I(\omega,\mathbf{k}) &= \sum_n |\langle\psi_n|c_{\mathbf{k},I}|\psi_0\rangle|^2\,\delta(\omega+E_n-E_0), \\ &= \sum_n \left|\langle\psi_n|\frac{c_{\mathbf{k},\alpha}+ic_{\mathbf{k},\alpha'}}{\sqrt{2}}|\psi_0\rangle\right|^2 \delta(\omega+E_n-E_0), \\ &= \frac{1}{2}\left[A_\alpha(\omega,\mathbf{k})+A_{\alpha'}(\omega,\mathbf{k})-if_{\alpha\alpha'}(\omega,\mathbf{k})+if_{\alpha'\alpha}(\omega,\mathbf{k})\right]. \end{aligned} \tag{S8}$$

Then the non-diagonal spectral functions for $\omega < 0$ can be expressed as

$$f_{\alpha\alpha'} = A_+ + iA_I - \frac{1+i}{2}(A_\alpha + A_{\alpha'}). \tag{S9}$$

Similarly, the above formula also holds for $\omega > 0$.

B. Definition of topological invariants via Green's functions

For single-particle topological insulators and superconductors, the associated symmetry-protected topological phase classification theories have been well established based on the periodic table approach [1]. Correspondingly, given the system Hamiltonian in Bloch space,

$$H(\mathbf{k}) = \sum_{i,j} c_i^\dagger \mathcal{H}_{ij}(\mathbf{k}) c_j, \tag{S10}$$

where $c_i^\dagger$ (c_i) denotes the fermionic generation (annihilation) operator on lattice site i. Then the topological invariants defined for labeling those topological phases can be constructed from the momentum space Hamiltonian matrix $\mathcal{H}(\mathbf{k})$ (or Bloch wavevectors $|\psi(\mathbf{k})\rangle$), which defines a map from the momentum space $\mathbf{k}$ to the space of matrix $\mathcal{H}(\mathbf{k})$.

For topological insulators with strong interactions, the topological invariants can no longer be simply described by those matrices like $\mathcal{H}(\mathbf{k})$ in Eq. (S10). Alternatively, one can utilize the single-particle retarded or advanced Green's function to construct topological invariants for both the non-interacting and interacting systems. This approach is inspired by the early works of Volovik [2], focusing on the two-dimensional topological superconductor model in class D. There, the zero-energy excitations are present at the shared boundary of two topologically distinct regions with the topological invariants defined via the single-particle Green's function in the frequency-momentum space, as

$$N_2 = \frac{1}{24\pi^2}\int d\Omega d^2k \mathrm{Tr}\left[\epsilon^{abc} G\partial_a G^{-1} G\partial_b G^{-1} G\partial_c G^{-1}\right], \tag{S11}$$

where $G \equiv G(i\omega,\mathbf{k})$ denotes the time-ordered Green's function in frequency-momentum space and a, b, $c \in \{\Omega, k_x, k_y\}$, with $\Omega = i\omega$ denoting the imaginary Matsubara frequency.

In this work, we take the 1D interacting Su-Schrieffer-Heeger (SSH) topological insulator as an example, which takes the following form

$$\hat{H} = -\sum(t_1 c_{i,\uparrow}^\dagger c_{i,\downarrow} + t_2 c_{i,\uparrow}^\dagger c_{i+1,\downarrow} + h.c.) + U\sum_i (n_{i,\uparrow} - \frac{1}{2})(n_{i,\downarrow} - \frac{1}{2}). \tag{S12}$$

The above model possesses the chiral symmetry $\hat{\Sigma}$, *i.e.*, $\hat{\Sigma}\hat{H}\hat{\Sigma}^\dagger = \hat{H}$, with the symmetry operator $\hat{\Sigma} = \prod_j [c_{j,\uparrow}^\dagger + (-1)^j c_{j,\uparrow}][c_{j,\downarrow}^\dagger + (-1)^j c_{j,\downarrow}]$. Hence in the periodic boundary condition, the corresponding frequency-momentum single-particle Green's function also obeys the chiral symmetry $\mathcal{C}G(i\omega,k)\mathcal{C} = -G(-i\omega,k)$ [3], where the chiral operator $\mathcal{C}$ squares to 1. Together with the result that $G(i\omega,k)$ is non-singular for such interacting topological insulator, then one can construct the topological invariant ξ of the 1D interacting SSH model directly from the zero-frequency Green's function $G(0,k)$ [4]

$$\xi = \frac{1}{4\pi i}\int dk\, \mathrm{Tr}\left[\mathcal{C}G^{-1}(0,k)\partial_k G(0,k)\right]. \tag{S13}$$

In the non-interacting limit with $U = 0$, the single-particle Green's function $G(i\omega,k)$ reduces to

$$G(i\omega,k) = \left[i\omega - \mathcal{H}(k)\right]^{-1}, \tag{S14}$$

and the zero-frequency Green's function is nothing but the inverse of the SSH Hamiltonian matrix $\mathcal{H}^{-1}(k)$. In this sense, defining topological invariants of interacting topological insulators via the Green's function method is a natural extension of those for non-interacting ones via the single particle Hamiltonians or eigenvectors.

SII. NUMERICAL SIMULATIONS OF THE SPECTRAL FUNCTIONS

In our numerical simulations, we consider a finite-size lattice with the periodic boundary condition. To simulate the experiment, we add to H a harmonic trapping potential

$$V_{\text{trap}} = \frac{1}{2} m\kappa^2 \sum_{i,\sigma} d_i^2 c_{i,\sigma}^\dagger c_{i,\sigma}, \tag{S15}$$

where κ is the trapping frequency, m is the mass of the atom, and d_i is the distance from the center of the trap to the lattice site i. We use $\gamma_T = m\kappa^2 a^2/2$ to parametrize the relative strength of the trap with a denoting the lattice constant. In the simulations, the γ_T is set to be 0.1, and the chemical potential is set to be zero. The exact diagonalization method is used to numerically diagonalize the Hamiltonian and find the energy spectrum and corresponding eigenstates. After we have obtained the full eigenstates and eigenenergies, we compute the spectrum function using Eq. (S6). The creation (annihilation) operators in momentum space are obtained by a Fourier transformation

$$\begin{cases} c_{\mathbf{k},\sigma} = \frac{1}{\sqrt{L_x}} \sum_{\mathbf{r}} e^{-i\mathbf{k}\cdot\mathbf{r}} c_{\mathbf{r},\sigma}, \\ c_{\mathbf{k},\sigma}^\dagger = \frac{1}{\sqrt{L_x}} \sum_{\mathbf{r}} e^{i\mathbf{k}\cdot\mathbf{r}} c_{\mathbf{r},\sigma}^\dagger, \end{cases} \tag{S16}$$

where $L_x = N$ is the lattice size number. Since in a real experiment, we can only measure the spectrum functions at discretized frequency and momentum, we use

$$\frac{1}{2\pi i}\left(\frac{1}{\omega - i\delta + E_n - E_0} - \frac{1}{\omega + i\delta + E_n - E_0}\right) \tag{S17}$$

to approximate the delta function: $\delta\left(\omega - E_n - E_0\right)$. To calculate the topological invariant χ, we first use a discretized version of

$$G_{\alpha\alpha}(i\omega, \mathbf{k}) = \int d\omega' \frac{A_{\alpha\alpha}\left(\omega'\right)}{i\omega - \omega'} \tag{S18}$$

to calculate the diagonal terms of Green's function at zero frequency. Then we use

$$G_{\alpha\alpha'} = \frac{1}{2}\left[2G_{++} - G_{\alpha\alpha} - G_{\alpha'\alpha'} + i\left(2G_{\text{II}} - G_{\alpha\alpha} - G_{\alpha'\alpha'}\right)\right], \tag{S19}$$

to calculate the non-diagonal terms [$G_{\tau\tau}$ is obtained from $A_\tau(\omega, \mathbf{k})$ ($\tau = \alpha, \alpha', +,$ or I)]. After we obtained the whole Green's function at different momentum, χ can be obtained directly by a discretized version of Eq. (S13).

In Fig. S1, we show the values of topological invariants calculated from the discretized spectral functions for different numbers of lattice sizes N, both with and without the trapping strength γ_T in Eq. (S15). We find that the two curves for $\gamma_T = 0$ and $\gamma_T = 0.1$ almost coincide with each other, indicating that the trapping potential has a minimal effect on the results of the topological numbers. Besides, Fig. S1 shows that the numerically simulated winding number converges to the expected value $\xi = 1$ quickly with the increasing of the system lattice size $L_x = N$. Hence in real experiments with hundreds of lattice sites, it is reasonable to expect that the extracted topological invariants are much quantized.

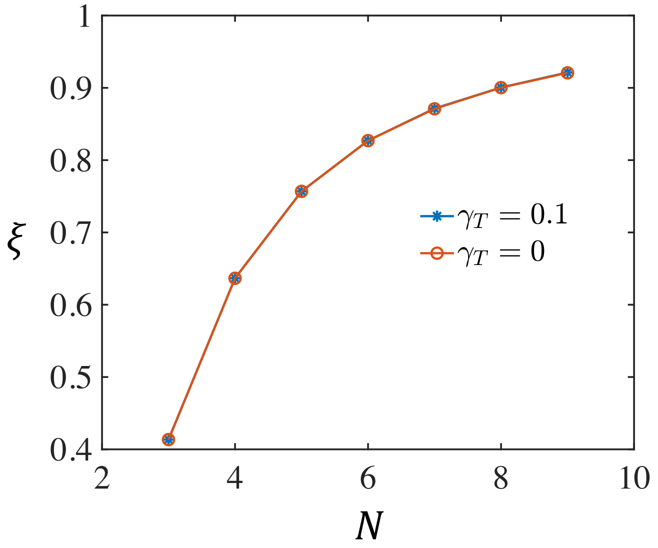

FIG. S1. The topological winding numbers ξ of the 1D interacting SSH model for different number of lattice sites N, including cases of both the trapping strength $\gamma_T = 0$ and $\gamma_T = 0.1$. Parameters: Interacting strength $U = 0.8$, spin-flip strength $t_1 = 0$, hopping strength $t_2 = 1$.

SIII. MORE DETAILS ABOUT THE UNSUPERVISED LEARNING OF INTERACTING TOPOLOGICAL PHASES

A. Learning non-interacting topological insulators based on spectral function: The 1D SSH model

The capability of diffusion map method in classifying phases of non-interacting topological insulators has been verified in previous work [5], where the momentum-space Hamiltonian or Bloch wavevectors are adopted as input data samples. To verify that Green's function, which is more achievable than those Bloch wavevectors in experiment, is also valid to be the input data for unsupervised machine learning based on the diffusion map method, here, we consider the non-interacting case of Eq. (S12) with $U = 0$, where the Hamiltonian becomes the 1D SSH model. We choose Green's functions as the input data set, *i.e.*, $\{\mathbf{x}^{(l)} | \mathbf{x}^{(l)} = [\hat{g}^{(l)}(0,k), k \in [-\pi, \pi)]\}$ by varying the spin flipping strength t_1 from 0 to 2, while fixing the hopping strength $t_2 = 1$, $U = 0$. $\hat{g}^{(l)}(0,k)$ can be obtained from the spectral functions $A(\omega, k)$. From the numerical results in Figs. S2(a-c), we show that the input samples are automatically clustered into two categories and the learned phase boundary locates at $t_1 \approx 1.01$, which is consistent with the phase boundary predicted by the values of the topological invariant in Fig. S2(d), as well as the theoretical phase boundary $t_1 = 1$. Here we label the red circle samples in Fig. S2(b) by the topological non-trivial phase, and the blue star samples in Fig. S2(b) by the topological trivial phase, respectively.

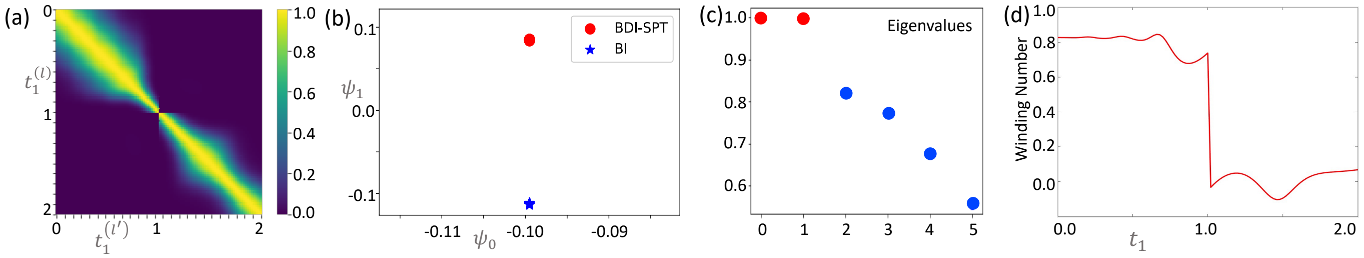

FIG. S2. Machine learning results of the 1D non-interacting topological phases with the data set of Green's function. (a) Heatmap of the Gaussian kernel value between the data samples with varying t_1. (b) Scatter diagram of the two eigenvectors $\{\psi_0, \psi_1\}$ of the diffusion matrix with the largest two eigenvalues $\lambda \approx 1$. The samples are clustered into two phases, where the red circle denotes the BDI-SPT phase, and the blue star denotes the band insulator (BI) phase. (c) The leading eigenvalues of the diffusion matrix, two eigenvalues $\lambda_{0,1} \approx 1$ are denoted by the red dots. (d) Winding number obtained from the numerically simulated spectral function with varying t_1. Parameters: number of sites $N = 6$, the variance parameter $\epsilon = 0.003$, $t_2 = 1$, $U = 0$, the varying parameter $t_1^{(l)} = 0.02 * (l-1)$ for each sample $\mathbf{x}^{(l)}$, with $l \in [1, 101]$.

B. More about the learning results of the 1D interacting topological insulator

In the main text, we have shown that the diffusion map method is valid in learning phase transition of topological insulators with strong interactions, from both analytical and numerical perspectives. For the 1D interacting SSH model we consider in this work, we successfully predict its phase boundary between the topological trivial (band insulator, abbreviated as BI) and non-trivial (BDI-SPT) phases, where BDI-SPT denotes the symmetry-protected topological phase in BDI symmetry class [1]. For the 1D interacting SSH model, besides the BDI-SPT and BI phases, it has been shown that there exists another phase called the *Aoki phase* [6], whose name is borrowed from the high-energy physics. The Aoki phase is a sort of symmetry-breaking phase. In the regime of 1D interacting SSH model, the Aoki phase occurs for sufficiently large U, which is also regarded as the density-wave phase with the non-zero local order parameter $\langle(n_{i,\uparrow} - n_{i,\downarrow})\rangle$. Nevertheless, by substituting the numerically simulated Green's function into Eq. (S13), one obtains the winding number $\xi \approx 0.7$ for the Aoki phase, see Fig. S3(d). This motivates us to classify the transition between the Aoki and BI phases based on the diffusion map method. In Fig. S3, we show the learning results of the interacting SSH model with $U = 4$. From the numerical results in Fig. S3(a-c), we show that the unsupervised learning method can classify the phase boundary between the Aoki and band insulator phases, with the phase boundary $t_1 \approx 0.202$.

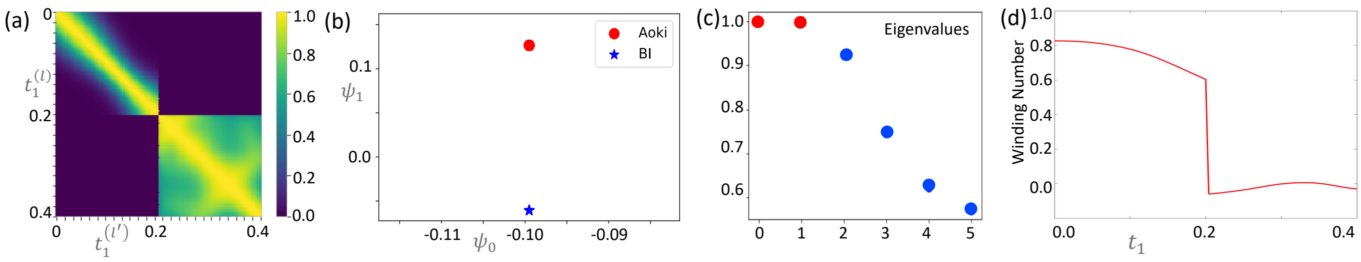

FIG. S3. Learning results of the 1D SSH model with strong interactions. Single-particle Green's function as the input data. Site number $N = 6$, the variance parameter $\epsilon = 0.003$, $t_2 = 1$, interacting strength $U = 4$, the varying parameter $t_1^{(l)} = 0.004 * (l - 1)$ for each sample $\mathbf{x}^{(l)}$, with $l \in [1, 101]$. (a) Heatmap of the Gaussian kernel value between the data samples with varying t_1. (b) Scatter diagram of the two eigenvectors $\{\psi_0, \psi_1\}$ of the diffusion matrix with the largest two eigenvalues $\lambda \approx 1$. The samples are clustered into two phases, where the red circle denotes the Aoki phase, and the blue star denotes the band insulator (BI) phase. (c) The leading eigenvalues of the diffusion matrix, two eigenvalues $\lambda_{0,1} \approx 1$ are denoted by the red dots. (d) Winding number obtained from the numerically simulated spectral function with varying t_1.

SIV. TOPOLOGICAL INVARIANTS FROM MOMENTUM-SPACE GREEN'S FUNCTION

In this section, we present the numerical results of the momentum space single-particle Green's function calculated based on the spectral functions. Here we show the trajectories of the values of inverse Green's functions $G^{-1}(0, k) = g_x\sigma_x + g_y\sigma_y + g_z\sigma_z$ in Brillouin zone, with a focusing on the topological winding numbers of such trajectories, *i.e.*, whether the corresponding trajectory envelops the original point. Fixing $t_2 = 1$, we consider three representative cases of the 1D model in Eq. (S12):

- Interacting strength $U = 0$. The model turns into the 1D SSH Hamiltonian, with a well-known critical point $t_1 = t_2$ in theory. Varying t_1 across the critical point would lead to the change of the topological invariant (winding number for 1D system), indicating the transition between topological non-trivial phase and topological trivial phase.

- Interacting strength $U = 1$. With the variation of t_1, the Hamiltonian is expected to behave a phase transition between topological non-trivial phase (BDI-SPT) and topological trivial phase (band insulator (BI)).

- Interacting strength $U = 4$. With the variation of t_1, the Hamiltonian is expected to behave a phase transition between Aoki phase and BI phase.

A. The non-interacting case: $U = 0$

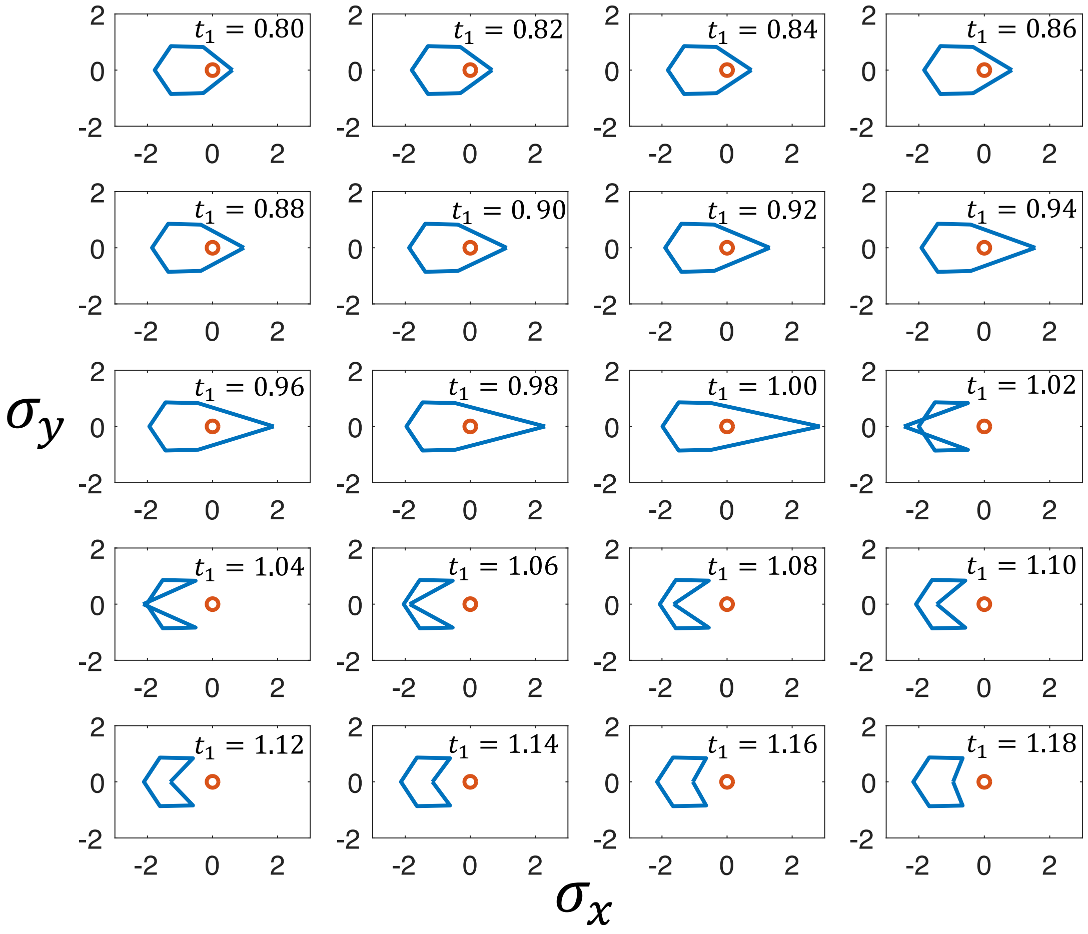

FIG. S4. 1D non-interacting SSH model ($U = 0, t_2 = 1$). Trajectory of the inverse of zero-frequency Green's function $G^{-1}(0, k) = \vec{g}(0, k) \cdot \vec{\sigma}$ in momentum space. The σ_z sector of $G^{-1}(0, k)$ is zero, *i.e.*, $g_z = 0$. Here we list the value distribution of g_x and g_y in $\{\sigma_x, \sigma_y\}$-plane. The blue lines represent the trajectories of $G^{-1}(0, k)$ in discrete first Brillouin zone $k \in \{\frac{2\pi}{N} * j | j \in \mathbb{Z}_N\}$, where N represents the site number of the model. Here we set $N = 6$. The red circle denotes the original point. With the variation of t_1 in step 0.02, the winding number of the inverse Green's function (blue trajectory) around the original point (red dot) would change from 1 to 0. The transition point lies in the interval $t_1 \in (1.00, 1.02)$, which matches exactly with our unsupervised learning result.

B. The interacting case: $U = 1$

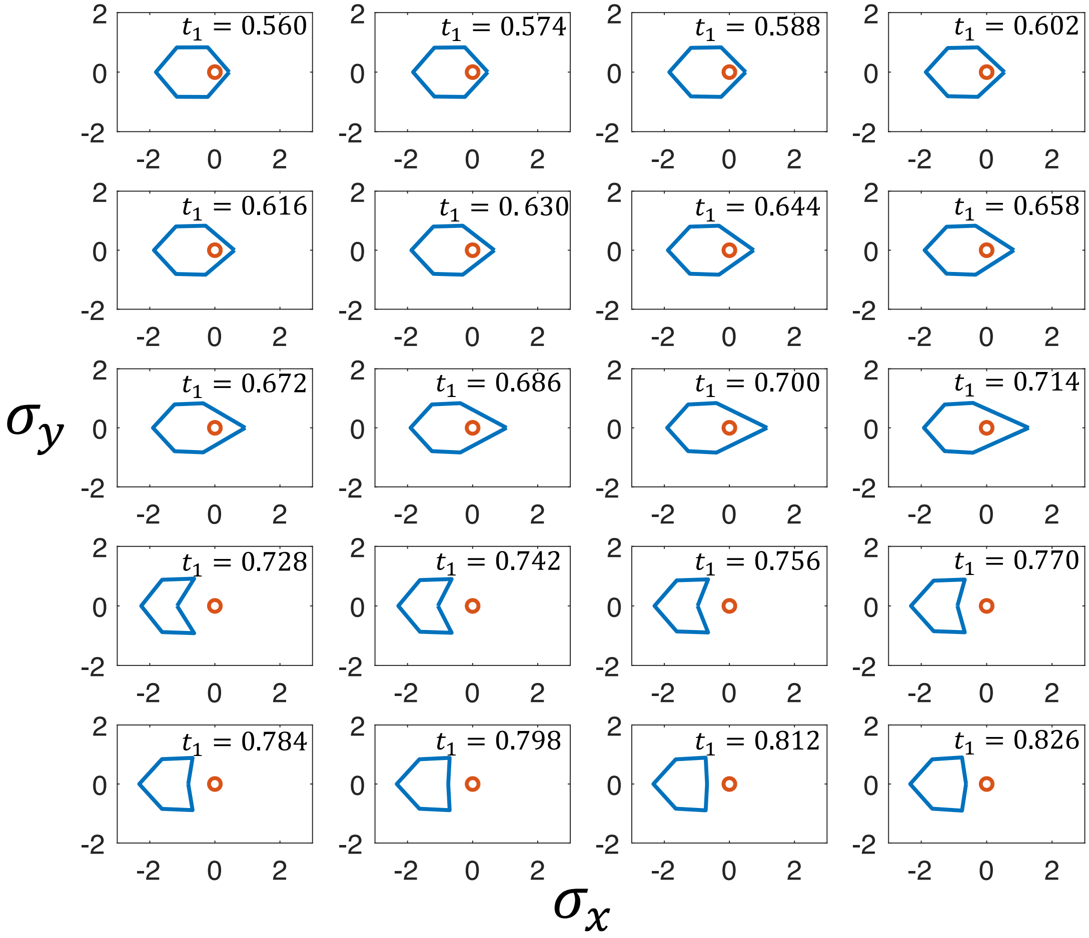

FIG. S5. 1D interacting SSH model ($U = 1$, $t_2 = 1$). Trajectory of the inverse of zero-frequency Green's function $G^{-1}(0, k) = \vec{g}(0, k) \cdot \vec{\sigma}$ in momentum space. The σ_z sector of $G^{-1}(0, k)$ is zero, *i.e.*, $g_z = 0$. Here we list the value distribution of g_x and g_y in $\{\sigma_x, \sigma_y\}$-plane. The blue lines represent the trajectories of $G^{-1}(0, k)$ in discrete first Brillouin zone $k \in \{\frac{2\pi}{N} * j | j \in \mathbb{Z}_N\}$, where N represents the site number of the model. Here we set $N = 6$. The red circle denotes the original point. With the variation of t_1 in step 0.014, the winding number of the inverse Green's function (blue trajectory) around the original point (red dot) would change from 1 to 0. The transition point lies in the interval $t_1 \in (0.714, 0.028)$, which matches exactly with our unsupervised learning result.

C. The interacting case: $U = 4$

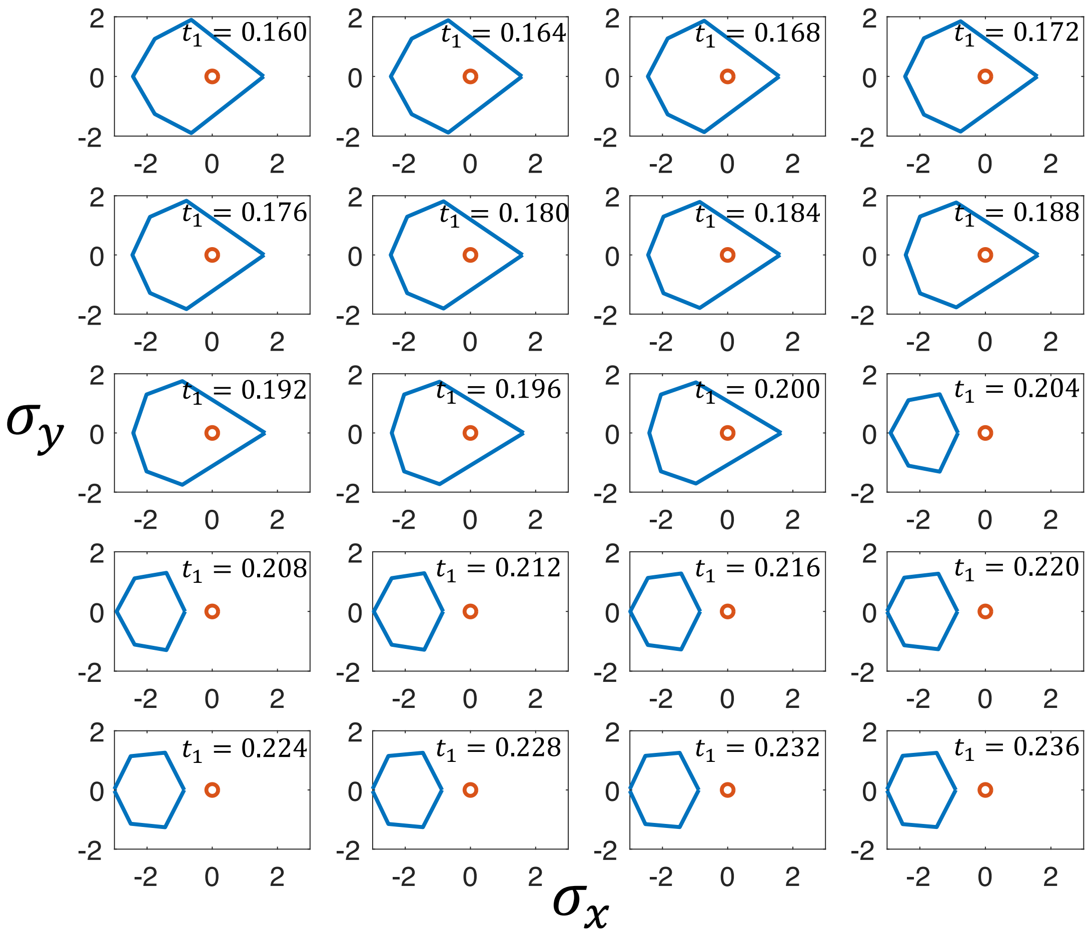

FIG. S6. 1D interacting SSH model ($U = 4$, $t_2 = 1$). Trajectory of the inverse of zero-frequency Green's function $G^{-1}(0,k) = \vec{g}(0,k) \cdot \vec{\sigma}$ in momentum space. The σ_z sector of $G^{-1}(0,k)$ is zero, *i.e.*, $g_z = 0$. Here we list the value distribution of g_x and g_y in $\{\sigma_x, \sigma_y\}$-plane. The blue lines represent the trajectories $G^{-1}(0,k)$ in discrete first Brillouin zone $k \in \{\frac{2\pi}{N} * j | j \in \mathbb{Z}_N\}$, where N represents the site number of the model. Here we set $N = 6$. Red circle denotes the original point. With the variation of t_1 in step 0.004, the winding number of the inverse Green's function (blue trajectory) around the original point (red dot) would change from 1 to 0. The transition point lies in the interval $t_1 \in (0.200, 0.204)$, which matches exactly with our unsupervised learning result.

SV. INTRODUCTION TO THE DIFFUSION MAP METHOD

The diffusion map method [7–9] provides a general framework that can non-linearly reduce the higher dimensional data samples into the lower ones, while preserving their prominently geometrical or topological structures. With the dimension-compressed data set, one can then cluster the unlabelled samples into categories in an unsupervised manner, without any prior knowledge about the system. We can understand the diffusion map method in the context of physics, *i.e.,* it combines the random walk Markov chain with the heat diffusion process. Now we demonstrate how to utilize the diffusion map method for dimensional reduction. Concretely, when we have a set of input samples, $\mathbf{x} = \{\mathbf{x}^{(1)}, \mathbf{x}^{(2)}, \cdots \mathbf{x}^{(L)}\}$, where $\mathbf{x}^{(i)}$ represents the i-th data sample with dimension d, *i.e.*, $\mathbf{x}^{(i)}$ is a point in complex space $\mathbb{C}^d$. Then one can define the connectivity (semi-positive and symmetric to) between arbitrary two samples $\mathbf{x}^{(l)}$ and $\mathbf{x}^{(l')}$ through, for example, the Gaussian kernel function

$$\mathcal{K}_{l,l'} = \exp\left(-\frac{\|\mathbf{x}^{(l)} - \mathbf{x}^{(l')}\|^2_{\mathbb{L}_p}}{2\epsilon}\right), \tag{S20}$$

where $\|\cdot\|_{\mathbb{L}_p}$ represents the $\mathbb{L}_p$-norm of the vectors, the parameter ϵ represents the Gaussian variance to be adjusted. In this work, we mainly focus on the case of $p = 2$, and the distance $\|\cdot\|_{\mathbb{L}_2}$ is the familiar Euclidean distance. Then through the Gaussian kernel, one can express the one-step diffusion probability between the two samples $\mathbf{x}^{(l)}$ and $\mathbf{x}^{(l')}$ as follows

$$\mathcal{P}_{l,l'} = \frac{\mathcal{K}_{l,l'}}{\sum_{l'} \mathcal{K}_{l,l'}}, \tag{S21}$$

where $\mathcal{P}_{l,l'}$ obeys the constraint $\sum_l \mathcal{P}_{l,l'} = 1$, which simply indicates that the total diffusion probability from the point $\mathbf{x}^{(l')}$ to other points is 1. Here $\mathcal{P}$ can be also interpreted as the one-step Markovian random walk matrix. Then after the $2t$ steps of random walk in the complex space $\mathbb{C}^d$, the final connectivity between two points $\mathbf{x}^{(l)}$ and $\mathbf{x}^{(l')}$ can be represented by the corresponding $2t$-step diffusion distance, as

$$D_t^2(l,l') = D_t^2(\mathbf{x}^{(l)}, \mathbf{x}^{(l')}) = \sum_{m=1}^{L} \frac{\left(\mathcal{P}^t_{l,m} - \mathcal{P}^t_{l',m}\right)^2}{\sum_j \mathcal{K}_{m,j}} = \sum_{k=1}^{L-1} \lambda_k^{2t} [(\psi_k)_l - (\psi_k)_{l'}]^2 \geq 0, \tag{S22}$$

where $\{\lambda_k | k \in [1, L-1]\}$ are the full eigenvalues of the one-step diffusion matrix $\mathcal{P}$, the eigenvalues rank in descending order, *i.e.*, $\lambda_0 = 1 \geq \lambda_1 \geq \cdots \geq \lambda_{L-1}$. The set of states $\{\psi_k | k \in [1, L-1]\}$ are the corresponding right eigenvectors of $\mathcal{P}$. The largest eigenvalue $\lambda_0 = 1$ does not contribute to the diffusion probability, since the corresponding right eigenvector ψ_0 is a constant vector with all elements equivalent owing to the diffusion probability conservation, *i.e.*, independent of the concrete formula of $\mathcal{P}$.

Actually, the $2t$-step diffusion distance $D_t(l,l')$ between $\mathbf{x}^{(l)}$ and $\mathbf{x}^{(l')}$ in Eq. (S22) has a Euclidean distance formula $D_t^2(\mathbf{x}^{(l)}, \mathbf{x}^{(l')}) = \|\Psi_t^{(l)} - \Psi_t^{(l')}\|^2_{\mathbb{L}_2}$ under the mapping

$$\mathbf{x}^{(l)} \to \Psi_t^{(l)} := [\lambda_1^t (\psi_1)_l, \lambda_2^t (\psi_2)_l, \cdots, \lambda_{L-1}^t (\psi_{L-1})_l]. \tag{S23}$$

Together with the fact that the eigenvalues obey $\{0 \leq \lambda_k \leq 1 | \forall k \in [1, L-1]\}$, after $t \to \infty$ steps, only the first few components with largest $|\lambda_k| \approx 1$ are dominant due to the exponentially decreasing term $(\lambda_k)^t$ with respect to t. Besides, the number of eigenvalues $\lambda_k \approx 1$ is equivalent to the number of blocks that the Gaussian kernel matrix is divided into. As a consequence, almost all the distance information about the original data set $\mathbf{X}$ is encoded in such few components. Then the original samples $\mathbf{x}^{(l)}$ with higher feature dimension are reduced to the lower ones in Euclidean space. With the compressed data in Ψ space, one can then apply the clustering method (e.g. k-means) to cluster the corresponding samples without any *a priori* knowledge. In classifying the topological phases of matter, the number of $|\lambda_k| \approx 1$ equals the number of topological clusters without prior labels, which can also be straightforwardly observed from the diagonal blocks of the Gaussian kernel matrix, see Fig. S2(a) for demonstration. Hence we conclude that the diffusion map method provides a possible approach to identifying different topological phases.

[1] A. Kitaev, Periodic table for topological insulators and superconductors, AIP Conf. Proc. **1134**, 22 (2009).
[2] G. E. Volovik, *The Universe in a Helium Droplet*, Vol. 117 (Oxford University Press, 2003).
[3] S. R. Manmana, A. M. Essin, R. M. Noack, and V. Gurarie, Topological invariants and interacting one-dimensional fermionic systems, Phys. Rev. B **86**, 205119 (2012).

[4] Z. Wang and S.-C. Zhang, Simplified topological invariants for interacting insulators, Phys. Rev. X **2**, 031008 (2012).
[5] M. S. Scheurer and R.-J. Slager, Unsupervised Machine Learning and Band Topology, Phys. Rev. Lett. **124**, 226401 (2020).
[6] Y. Kuno, Phase structure of the interacting Su-Schrieffer-Heeger model and the relationship with the Gross-Neveu model on lattice, Phys. Rev. B **99**, 064105 (2019).
[7] R. R. Coifman, S. Lafon, A. B. Lee, M. Maggioni, B. Nadler, F. Warner, and S. W. Zucker, Geometric diffusions as a tool for harmonic analysis and structure definition of data: Diffusion maps, Proc. Natl. Acad. Sci. **102**, 7426 (2005).
[8] R. R. Coifman, S. Lafon, A. B. Lee, M. Maggioni, B. Nadler, F. Warner, and S. W. Zucker, Geometric diffusions as a tool for harmonic analysis and structure definition of data: Multiscale methods, Proc. Natl. Acad. Sci. **102**, 7432 (2005).
[9] R. R. Coifman and S. Lafon, Diffusion maps, Appl. Comput. Harmon. Anal. **21**, 5 (2006).
